# Pygidial Glands in Carabidae, an Overview of Morphology and Chemical Secretion

**DOI:** 10.3390/life11060562

**Published:** 2021-06-15

**Authors:** Anita Giglio, Maria Luigia Vommaro, Pietro Brandmayr, Federica Talarico

**Affiliations:** 1 Department of Biology, Ecology and Earth Science, University of Calabria, 87036 Rende, Italy; marialuigia.vommaro@unical.it (M.L.V.); pietro.brandmayr@unical.it (P.B.); 2 Natural History Museum and Botanical Garden, University of Calabria, 87036 Rende, Italy; federica.talarico@unical.it

**Keywords:** allomone, chemical ecology, defensive secretion, gas chromatography, ground beetles, microscopy, morphology

## Abstract

Predator community structure is an important selective element shaping the evolution of prey defence traits and strategies. Carabid beetles are one of the most diverse families of Coleoptera, and their success in terrestrial ecosystems is related to considerable morphological, physiological, and behavioural adaptations that provide protection against predators. Their most common form of defence is the chemical secretion from paired abdominal pygidial glands that produce a heterogeneous set of carboxylic acids, quinones, hydrocarbons, phenols, aldehydes, and esters. This review attempts to update and summarise what is known about the pygidial glands, with particular reference to the morphology of the glands and the biological function of the secretions.

## 1. Introduction

The carabid beetles (Coleoptera, Carabidae) include approximately 40,000 described species that are ecologically important as predators in many ecosystems and range in feeding habits from generalist to specialists [1,2]. Carabids are often used as indicators because they are extremely sensitive to environmental changes [3,4,5]. Their ecological role in the trophic web of agroecosystems [6], makes them particularly suitable for monitoring the impact of agrochemicals [5,7,8] and heavy metals [9,10,11,12,13]. Furthermore, as generalist predators, ground beetles provide important ecosystem services by lowering populations of invertebrate pests and weed seeds [14,15]. However, carabids are consumed by a number of different species, including invertebrates and insectivorous vertebrates such as birds, mammals, amphibians, and reptiles [1]. Predator–prey interactions are likely the major driving force for the evolution of defences against predators in carabid beetles. Strategies to escape predatory attacks primarily include morphological adaptations, such as cryptic or warning coloration [16,17,18,19] and dorso-ventral flattening, large eyes, and long legs to escape [20], as well as secretion of chemical repellents [21,22,23]. Ground beetles possess a pair of abdominal glands called pygidial glands that produce defensive secretions. The main function of the pygidial glands is to defend against predators, but they also engage in biological activities such as facilitating the penetration of the defensive substances into the integument of the predator and inhibiting the growth of fungi and pathogens [24,25]. A few studies to date have examined the chemical compounds of pygidial gland secretions [26,27,28,29,30] and comparatively investigated their taxonomic significance [22,31,32,33,34]. We attempt to review the current state of knowledge on the pygidial glands of carabid beetles by providing an overview of their structure and the chemical compounds of the secretion.

## 2. General Morphology

Forsyth [35] first proposed a comparative description of pygidial glands in 71 species from 34 tribes to define phylogenetic relationships within Carabidae. Currently, approximately 150 species from 43 tribes have been described (Table 1). The most commonly used examining technique to study pygidial gland morphology is light microscopy (LM). In addition, other techniques such as fluorescence (FM) microscopy, scanning electron microscopy (SEM) and focused ion beam/scanning (FIB/SEM) electron microscopy, (TEM) transition electron microscopy, synchrotron radiation X-ray phase-contrast micro-tomography (SR-PhC micro-CT) are also applied.

**Figure 1:**
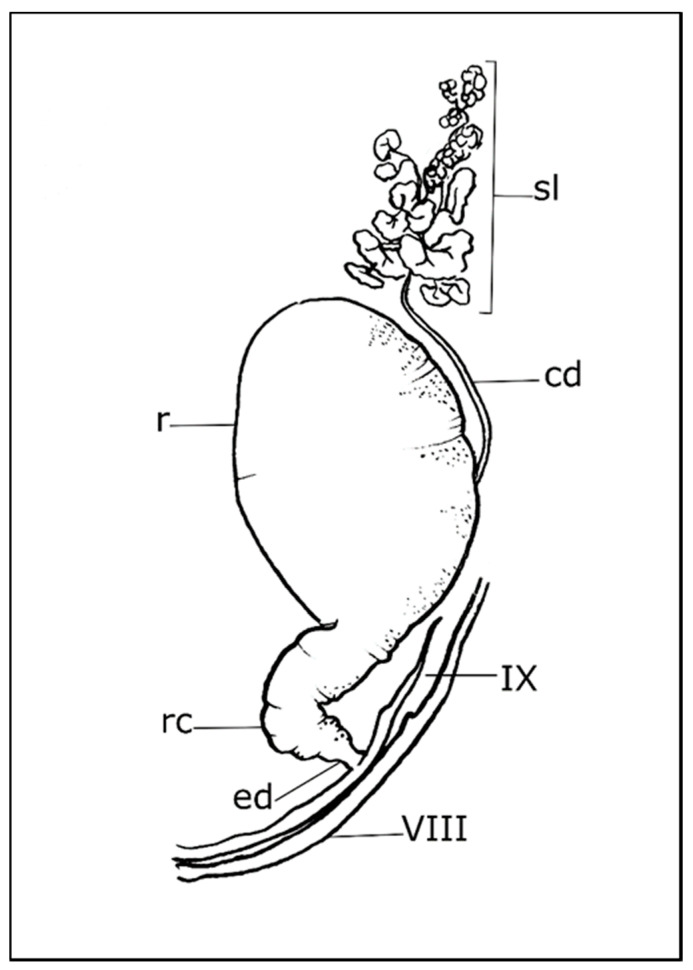
Schematic drawing of a pygidial gland. cd: collecting duct; ed: efferent duct; r: reservoir chamber; rc: reaction chamber; sl: secretory lobe; VIII: eighth tergite; IX: ninth tergite (for more details of species listed in the text, see Forsyth (1972) [35]).

Each pygidial gland consists of a variable number of secretory lobes (acini), collecting duct, reservoir chamber, reaction chamber, and efferent duct (Figure 1). These glands (class 3 according to the classification of Noirot and Qhennedey [36,37]) are variable in structure and have been described in several species [35,38]. The lobe or acinus, which is spherical or elongated and enveloped in a thin basal lamina, is a cluster of secretory units, connected to the collecting duct by a conducting duct that drains secretions outward. The secretory unit consists of two parts, an elongated, cube-shaped secretory cell surrounding a receiving duct and a duct cell surrounding the conducting duct [35,39,40,41]. The receiving duct is a porous tube composed of one or more layers of epicuticle located in its extracellular space and bounded by microvilli. The collecting duct has an epithelial wall of flattened cells, lined by endocuticle, and a thin layer of epicuticle that is regularly folded into spiral ridges, annular arrays, or pointed peg-like projections, that reduce the volume of the lumen to control the free flow of secretion to the reservoir chamber [35,39,41]. The entrance of the collecting duct to the reservoir chamber is of great variability. It is located at the anterior or middle position in Scaritinae, Brachininae, and some Bembidiini, Pterostichini, Amarini, Carabini, Nebriini, Metriini, and Paussini [33,35,39,40,42,43,44]. While it is located near the entrance of the efferent duct in Harpalini, Agonini, Chlaeniini, Dryptini, Anthiini, Lebiini, Trachypachini, Omophronini, Loxandrini, Catapieseini, Galeritini, and Zuphini [31,33,35,45]. The reservoir chamber is a spherical, elongate, or bilobate compartment of variable size. Interwoven muscle bundles cover the outer wall and are connected to tracheal branches. The basal membrane supports flattened epithelial cells covered by a thin uniform layer of endo- and epicuticle. The muscular contraction regulates secretion through a valve that separates the reservoir from the reaction chamber. In Paussinae, Brachininae, and Carabinae, an accessory gland is located below the valve [35]. Secretions from the reservoir chamber are mixed with secretions from the accessory glands in the reaction chamber. The efferent duct leads from the reservoir chamber to the external orifice. The close association of the pygidial glands with the tracheal branches suggests a high aerobic metabolism.

The external orifice is located dorso-laterally in the posterior part of the abdomen, near to the antero-lateral margin of the ninth tergite, and close to the tergo-sternal suture in Carabinae, Scaritinae, Paussinae, Elaphrinae, Broscinae, and Brachinini, or at the posterolateral margin of the eighth tergite in derived lineages, e.g., Trechinae and Harpalinae, and including Licinini, Chlaeniini, Panagaeini, Anthiini, Zabrini, Oodini, Pterostichini, and Agonini [35,46]. Differences in pygidial gland morphology between sexes have been reported in *Cicindela campestris* [47].

## 3. Excretory Mechanisms

Oozing, spraying, and crepitation are the main types of external excretory mechanisms observed in carabid beetles in response to disturbance [58]. Oozing of secretion over the cuticle of the hind segments occurs in species that have relatively weakly developed muscles on the wall of the reservoir chamber, i.e., in the tribes Nebriini, Notiophilini, Loricerini, Elaphrini, and the subfamilies Scaritinae, Cicindelinae, and Broscinae [32,35]. This is probably the plesiomorphic mode of discharge, whereas the secretion expelled by strong muscle pressure on the reservoirs is an apomorphic adaptation. The discharge of a directional secretion by turning the tip of the abdomen has been observed in many taxa that exhibit a variable secretion discharge, such as Trechini, Bembidiini, Galeritini, Carabini, Cychrini, Harpalini, Agonini, Anthiini, and Pterostichini (except the genus *Abax*) [32,45,59]. Bombardier beetles discharge secretion by crepitation [60,61], with the exception of *Metrius contractus*, which discharges its secretion using the oozing ancestral discharge mechanism [39,62]. This discharge has evolved independently in Ozaenini and Paussini on the one hand and in Brachinini on the other. In the tribe Brachinini, the explosive defence is an active enzymatic exothermic reaction that produces benzoquinones, free oxygen, water, and heat up to 100 °C [55]. The process begins with muscle contraction of the reservoir chamber, which allows stored hydroquinones and hydrogen peroxide, to move through the one-way valve, enter the reaction chamber, and mix with catalases and peroxidases produced by the accessory glands. In Paussinae, fluids are directed via a cuticular fold (Coanda flange) located at the posterolateral angle of the elytra, which serves as a launching guide for rapid anterior discharge [60,61,63]. The ability to direct the sprayed secretion has also been observed in *Calosoma prominens* [64].

## 4. Chemical Compounds of Secretion

To date, over 363 species from 45 tribes have been studied by gas chromatography-mass spectrometry (GC-MS) (Table 2) in dichloromethane or hexane extracts. The semiochemicals, listed in Table 2, belong to one of the following classes: aliphatic and aromatic carboxylic acids, phenols (m-cresol and xylenol), aldehydes, quinones, hydrocarbons, ketones, terpenes, and esters. The biosynthetic pathways of these compounds have been extensively studied in arthropods [27,65]. However, studies addressing their biogenesis in the pygidial gland of carabids are lacking. The enzymatic derivation of quinones is one of the few metabolic pathways investigated. The bombardier beetle *Brachinus elongatulus* has the ability to convert m-cresol to 2-methyl-1,4-hydroquinone, which is then oxidised to 2-methyl-1,4-benzoquinone (toluquinone), within 24 h in its defensive spray, when added to food or injected into the haemocoel [66]. An origin from amino acids has been demonstrated for carboxylic acids. Valine is converted into methacrylic and isobutyric acids in *Carabus taedatus* [67] and *Scarites subterraneus* [68]. Biosynthesis of both tiglic and ethacrylic acid from isoleucine via 2-methylbutyric acid has been demonstrated in *Pterostichus californicus* [69]. Indeed, valine and isoleucine are essential amino acids, diet-dependent and strictly regulated by the availability of resources [70].

### 4.1. Interspecific Adaptations

The chemical composition of pygidial gland secretions exhibits interspecific variability within and among subfamilies (Table 2). This variability is the result of a trade-off between the diversity of predators in different habitats and the fitness costs of resource allocation in life traits such as behavioural defences against these enemies [2,71].

The chemicals found in secretions belong to two different functional categories, allomones and bacteriostats. Allomones are primarily involved in the secondary antipredator responses that carabids exhibit as prey to actively defend themselves against predators. Ground beetles emit volatile substances directed at specific groups of arthropods or vertebrates that act as repellents on the chemoreception of predators or interfere with physiological processes as irritants (emesis, vesication) [58]. Deterrent, toxic, and irritant properties of pygidial gland secretions are known in bombardier beetles (Brachinini), which release irritant quinones by a hot, pulsed spray mechanism [55,61,66] as an antipredator defence [72,73]. Quinones are the main class of compounds also found in the secretions of obligate or facultative myrmecophilous species belonging to Metrini, Ozaenini, and Paussini [34,74]. In the defensive secretions of Clivinini [75] and *Metrius contractus* [62], they are associated with complex mixtures of monoterpenes or hydrocarbons (Table 2). Saturated and unsaturated aliphatic carboxylic acids and fatty acids are widely distributed in the subfamilies Carabinae, Loricerinae, Nebrinae, Scaritinae, Scaritinae, and Harpalinae. They are recorded as a separate compound class in some species that belong to the tribes Pamborini, Elaphrini, Loricerini, Omophronini, Notiophilini, Broscini, Patrobini, Sphodrini, Pterostichini, Platynini, Harpalini, Licinini, and Lebiini. In Cychrini, irritant carboxylic acids (i.e., methacrylic acid) and fatty acids (i.e., tiglic acid) are released, associated with a stridulatory elytra-abdominal mechanism acting as an acoustic warning signal against predators [76,77]. Behavioural analyses showed that *Pasimachus subsulcatus* (Scaritinae) secretes a mixture of methacrylic acid and fatty acids to protect itself from lizards [51,52]. Carboxylic acids are also found in variable associations with terpenes, quinones, and hydrocarbons in Trechinae, and Harpalinae (Table 2). The repellent effect of salicylaldehyde in the secretion of *Calosoma prominens* has been tested against ants and vertebrates [64]. This chemical has also been detected in *C. sycophanta, C. schayeri, C. oceanicum* [34,48], *C. prominens* [64], *C. chinenses* [33], *Loxandrus longiformis* [34], and *Bembidion quadriguttatum, A. flavipes* [78] in a mixture with carboxylic acids. Benzaldehyde is the typical component of secretion in Cicindelinae [79,80]. It is produced via a cyanogenetic pathway that is absent in the other carabid subfamilies [81]. In tiger beetles, secretion of benzaldehyde may be associated with several antipredator characters, including aposematic camouflage, flight, and gregarious behaviour to avoid predators such as robber flies, lizards, and birds [17,82,83].

Synergism between polar volatile irritant compounds and lipophilic components of secretion has been demonstrated. Nonpolar lipophilic components from *Galerita lecontei* (long-chain hydrocarbons and esters) act as wetting and penetration enhancers and facilitate the spread of volatile polar compounds such as formic and acetic acids in the cuticle of predators [45]. In *Helluomorphoides clairvillei*, n-nonyl acetate facilitates the spread of formic acid through the epidermis or cuticle of predators [84]. The same surfactant effect has been attributed to hydrocarbons [85] for the uptake of repellent quinones in *Metrius contractus* [62].

The mixture of substances in glandular secretions also has biological functions. In vitro assays have shown that the pygidial gland secretion inhibits cell proliferation [86]. The mixture of aromatic (benzoic acid) and aliphatic carboxylic acids, esters, and terpenes have antimicrobial and fungicidal activity in *Carabus ullrichii, C. coriaceus*, *Abax parallelepipedus* [49], caterpillar hunter *Calosoma sycophanta* [48], and troglophilic and guanophilic *Laemostenus (Pristonychus) punctatus* [87]. Complex mixtures of monoterpenes are found in the defensive secretions of a large number of the species reported here (Table 2). Terpenes are volatile and are present in glandular secretions of many taxa, acting as chemical deterrents, trail scents, mating attractants, or alarm pheromones [22]. In carabid beetles, they have also been detected in the pupal stages of *Carabus lefebvrei* [88].

### 4.2. Intraspecific Adaptations

Little is known about intraspecific variation in secretion as a function of sex, age, and resource availability. Nevertheless, data collected to date suggest that chemical secretion plays a parsimonious role in both antipredation and mating behaviour. In *Oodes americanus,* defensive secretion shows qualitative differences in males and females [89]. The sexual dimorphism of carboxylic acids found in the defensive secretion of *Chlaenius cordicollis* depends on the reproductive status and age of both sexes and provides a means of chemical communication between the sexes [89,90]. Sex-specific variation likely protects mates during copulation, and the flower number of compounds in female secretions saves females the cost of synthesising them [90]. Although compounds that act as pheromones, such as pentacosadiene, 7-hexyldocosane, 9-methyltetracosane, have been detected in *Laemostenus punctatus, Trachypachus gibbsi,* and *Helluomorphoides clairvillei,* studies on their role in alarm or sex-aggregation reproductive behaviour are limited [27].

Intra- and inter-population variation in defensive secretion has also been documented to reflect genetic variability at the population level in responses to selective habitat pressure, as observed in *Chaenius cordicollis* [91], *Pasimachus subsulcatus* [51], and Cicindelinae [80]. On the other hand, the shift in secretion composition may have a dietary origin, as observed in species of the genus *Scaphinotus* [77]. These findings suggest the role of dietary chemical precursors in the biosynthesis of chemical secretions.

## 5. Concluding Remarks

Pygidial glands are homologous structures in the Carabidae. They show a range of morphological variations in structural elements, i.e., number of acini, the morphology of ducts and reservoir chamber, and mode of secretion discharge, among carabid species, regardless of habitat and associated ecological differences. Chemical defences are an important part of antipredator strategies in ground beetles. Prey–predator coevolution likely influences glandular secretion composition, which is the result of a trade-off between the predator diversity and the fitness costs of defending against these enemies. A great deal of interspecific diversity in the distribution of substances has been found in subfamilies. Some chemicals are readily identifiable as specific to particular taxa, while others show great species-level diversity among genera or tribes. These results are broadly consistent with previous studies in which the taxonomic distribution of compound secretion was reviewed according to habitat diversification and by mapping chemical classes in a phylogenetic context [31,33]. However, some elements need to be considered in the future interpretation of the taxonomic distribution of chemicals. The findings pertain to only the 4% of carabid species so far described, and further studies are needed to clarify differences in chemical composition in additional taxa. A large number of studies reported only the most abundant chemicals, neglecting compounds that are present in smaller percentages and have additional biological functions in the mixture, e.g., surfactants, pheromones, and antiseptic agents. In addition, the differences found in some chemical profiles may be related to the number of samples analysed as single or mixed samples or to the accuracy of the gas chromatographic equipment used in early studies. Finally, we recommend that further research should address to elucidate: (1) the biogenesis of all chemicals described in the pygidial glands and their function in an ecological context; (2) clarify the phylogenetic distribution patterns of chemicals by studying as many species as possible using comparable protocols; (3) the sexual dimorphism of the secretion with regard to the different degree of resource allocation between the sexes under the pressure of environmental selection.

## Figures and Tables

**Table 1:** Summary of carabid species in which the pygidial gland morphology has been investigated and the method used for analyses. Abbreviations—CLSM: confocal laser scanning microscopy; FIB/SEM: focused ion beam/scanning electron microscopy; FM: fluorescence microscopy; LM: light microscopy; NLM: non linear microscopy; SEM: scanning electron microscopy; SR-PhC micro-CT: synchrotron radiation X-ray phase-contrast micro tomography.

^§^ Subfamily	Tribe	Genus	Species	Methodology	Refs
**Paussinae**	**Metriini**	*Metrius*	*M. contractus*	LM; FIB/SEM	[35,39]
		*Sinometrius*	*S.* *turnai*	LM; FIB/SEM	[39]
	**Ozaeniini**	*Mystropomus*	*M. regularis*	LM	[34]
	**Paussini**	*Paussus*	*P. favieri*	LM; FM; FIB/SEM	[40]
			*P. laevifrons*	LM	[35]
		*Heteropaussus*	*H. jeanneli*	LM	[35]
**Cicindelinae**	**Cicindelini**	*Cicindela*	*C. campestris*	LM	[47]
			*C. hibrida*	LM	[47]
**Carabinae**	**Carabini**	*Calosoma*	*C. oceanicum*	LM	[34]
			*C. schayeri*	LM	[34]
			*C. senegalense*	LM	[35]
			*C. sycophanta*	LM	[48]
		*Carabus*	*C. (Tomocarabus) convexus*	LM	[42]
			*C. (Procustes) coriaceus*	LM	[42,49]
			*C. problematicus*	LM	[35]
			*C. ullrichii*	LM	[49]
			*C. (Megodontus) violaceus*	LM	[43]
	**Cychrini**	*Cychrus*	*C. caraboides rostratus*	LM	[35]
	**Pamborini**	*Pamborus*	*P. alternans*	LM	[34]
**Elaphrinae**	**Elaphrini**	*Elaphrus*	*E. cupreus*	LM	[35]
		*Blethisa*	*B. multipunctata*	LM	[35]
**Loricerinae**	**Loricerini**	*Loricera*	*L. pilicornis*	LM	[35]
**Omophroninae**	**Omophronini**	*Omophron*	*O. dentatum*	LM	[35]
**Nebriinae**	**Nebriini**	*Eurynebria*	*E. complanata*	LM	[35]
		*Leistus*	*L. ferrugineus*	LM	[35]
		*Nebria*	*N. brevicollis*	LM	[35]
			*N. psammodes*	LM	[50]
	**Notiophilini**	*Notiophilus*	*N. substriatus*	LM	[35]
**Scaritinae**	**Clivinini**	*Clivina*	*C. basalis*	LM	[34]
			*C. collaris*	LM	[35]
			*C. fossor*	LM	[35]
		*Schizogenius*	*S. lineolatus*	LM	[31]
	**Dyschiriini**	*Dyschirius*	*D. globosus*	LM	[35]
	**Pasimachini**	*Pasimachus*	*P. elongatus*	LM	[35]
			*P. subsulcatus*	LM	[51,52]
	**Carenini**	*Carenum*	*C. bonelli*	LM	[34]
			*C. interruptum*	LM	[34]
			*C. tinctillatum*	LM	[34]
		*Laccopterum*	*L. foveigerum*	LM	[34]
		*Philoscaphus*	*P. tuberculatus*	LM	[34]
**Broscinae**	**Broscini**	*Eurylychnus*	*E. blagravei*	LM	[34]
			*E. ollifi*	LM	[34]
		*Promecoderus*	*P.* sp.	LM	[34]
**Trechinae**	**Trechini**	*Thalassotrechus*	*T. barbarae*	LM	[35]
		*Trechus*	*T. obtusus*	LM	[35]
	**Bembidiini**	*Bembidion*	*B. lampros*	LM	[28,35]
			*B. rupestre*	LM	[35]
**Patrobinae**	**Patrobini**	*Amblytelus*	*A. curtus*	LM	[34]
		*Patrobus*	*P. longicornis*	LM	[31]
			*P. septentrionis*	LM	[35]
**Harpalinae**	**Morionini**	*Morion*	*M. simplex*	LM	[31]
		*Moriosomus*	*M. seticollis*	LM	[31]
	**Perigonini**	*Diploharpus*	*D. laevissimo*	LM	[31]
	**Loxandrini**	*Loxandrus*	*L. icarus*	LM	[31]
			*L. longiformis*	LM	[34]
			*L. velocipes*	LM	[31]
		*Oxycrepis*	*O.* sp.	LM	[31]
	**Sphodrini**	*Calathus*	*C. ambiguus*	LM	[35]
		*Pristonychus*	*P. terricola*	LM	[35]
	**Pterostichini**	*Abacomorphus*	*A. asperulus*	LM	[34]
		*Abaris*	*A. anaea*	LM	[31]
		*Blennidus*	*B. liodes*	LM	[31]
		*Castelnaudia*	*C. superba*	LM	[34]
		*Cratoferonia*	*C. phylarchus*	LM	[34]
		*Cratogaster*	*C. melas*	LM	[34]
		*Cyclotrachelus*	*C. sigillatus*	LM	[31]
		*Gasterllarius*	*G. honestus*	LM	[31]
		*Incastichus*	*I. aequidianus*	LM	[31]
		*Loxodactylus*	*L. carinulatus*	LM	[34]
		*Myas*	*M. coracinus*	LM	[31]
		*Notonomus*	*N. angusribasis*	LM	[34]
			*N. crenulatus*	LM	[34]
			*N. miles*	LM	[34]
			*N. muelleri*	LM	[34]
			*N. opulentus*	LM	[34]
			*N. rainbowi*	LM	[34]
			*N. scotti*	LM	[34]
			*N. triplogenioides*	LM	[34]
			*N. variicollis*	LM	[34]
		*Prosopogmus*	*P. harpaloides*	LM	[34]
		*Pseudoceneus*	*P. iridescens*	LM	[34]
		*Pterostichus*	*P. (Cophosus) cylindricus*	LM; NLM	[44]
			*P. (Monoferonia) diligendus*	LM	[31]
			*P. externepunctatus roccai*	LM	[50]
			*P. fortis*	LM	[33]
			*P. luctuosus*	LM	[31]
			*P. madidus*	LM	[38]
			*P. melanarius*	LM	[35]
			*P. melas*	SR-PHC MICRO-CT	[53]
			*P. (Pseudomaseus) nigrita*	LM; NLM	[44]
		*Rhytisternus*	*R. laevilaterus*	LM	[34]
		*Sarticus*	*S. cyaneocinctus*	LM	[34]
		*Sphodrosomus*	*S. saisseri*	LM	[34]
		*Trichosternus*	*T. nudipes*	LM	[34]
	**Platynini**	*Agonum*	*A. dorsale*	LM	[35]
	**Zabrini**	*Amara*	*A. aenea*	LM	[35]
		*Curtonotus*	*C. fulvus*	LM	[35]
		*Zabrus*	*Z. tenebriodes*	LM	[35]
	**Molopini**	*Abax*	*A. parallelepipedus (*sub*:A. ater)*	LM	[35,49]
		*Molops*	*M. (Stenochoromus)montenegrinus*	LM; NLM	[44]
	**Harpalini**	*Bradycellus*	*B. harpalinus*	LM	[35]
		*Diaphoromerus*	*D. edwardsi*	LM	[34]
			*H. aeneus*	LM	[35]
		*Harpalus*	*H. pensylvanicus*	CLSM	[54]
		*Pseudophonus*	*P. rufipes (sub:pubescens)*	LM	[35]
	**Licinini**	*Badister*	*B. bipustulatus*	LM	[35]
		*Dicrochile*	*D. brevicollis*	LM	[34]
			*D. goryi*	LM	[34]
		*Licinus*	*L. depressus*	LM	[35]
		*Syagonix*	*S. blackburni*	LM	[34]
	**Chlaeniini**	*Chlaenius*	*C. australis*	LM	[34]
			*C. cumatilis*	LM	[35]
			*C. inops*	LM	[33]
			*C. pallipes*	LM	[33]
			*C. velutinus*	LM	[50]
			*C. vestitus*	LM	[35,50]
		*Oodes*	*O. amaroides*	LM	[31]
			*O. hehpioides*	LM	[35]
	**Panagaenini**	*Craspedophorus*	*C.* sp.	LM	[34]
		*Panagaeus*	*P. crux-major*	LM	[35]
		*Psecadius*	*P. eustalactus*	LM	[35]
		*Tefflus*	*T.* sp.	LM	[35]
	**Masoreini**	*Masoreus*	*M. wetterhlii*	LM	[35]
	**Odacanthini**	*Colliuris*	*C. melanura*	LM	[35]
			*C. pensylvanica*	LM	[31]
	**Lebiini**	*Eudalia*	*E. macleayi*	LM	[34]
		*Metabletus*	*M. foveatus*	LM	[35]
		*Movmolyce*	*M. phyllodes*	LM	[35]
	**Galeritini**	*Galerita*	*G. lecontei*	LM	[45]
	**Anthiini**	*Anthia*	*A. artemis*	LM	[35]
	**Helluonini**	*Helluo*	*H. costatus*	LM	[34]
	**Dercylini**	*Dercylus (s.s.)*	*D.* sp.	LM	[31]
	**Catapieseini**	*Catapiesis*	*C. attenuata*	LM	[31]
			*C. sulcipennis*	LM	[31]
	**Dryptini**	*Drypta*	*D. dentata*	LM	[35]
			*D. japonica*	LM	[33]
	**Pseudomorphini**	*Sphallomorpha*	*S. colymbeioides*	LM	[34]
**Brachininae**	**Brachinini**	*Aptinus*	*A. bombarda*	LM; FM; FIB/SEM	[41]
			*A. crepitus*	LM; FM; FIB/SEM	[41]
			*A. displosor*	LM	[35]
		*Brachinus*	*B. crepitans*	LM	[35]
			*B. elongatus*	LM; FM; FIB/SEM; SEM	[41,55]
			*B. sclopeta*	LM; FM; FIB/SEM	[41]
			*B. stenoderus*	LM	[33]
			*P. verticalis*	LM	[34]
		*Pheropsophus*	*P. africanus*	LM; FM; FIB/SEM	[41]
			*P. hispanus*	LM; FM; FIB/SEM	[41]
			*P. lissoderus*	LM	[35]
			*P. occipitalis*	LM; FM; FIB/SEM	[41]
			*P. verticalis*	LM	[34]

^§^ Classification of taxa has been arranged according to Bousquet [56] and Beutel and Ribera [57].

**Table 2:** Components of pygidial gland secretions in Carabidae. Classification of taxa has been arranged according to Bousquet [56] and Beutel and Ribera [57].

Subfamily	Tribe	Genus	Species	Substances *	Refs
**Paussinae**	**Metriini**	*Metrius*	*M. contractus*	H18, H19, H20, H21, H22, H23, H25, H26, H27, H28, H29, H36, H35, H37, H41, H42, H52, H53, H54, H55, H56, H62, Q2, Q3, Q8, Q11, Q13	[62]
	**Ozaeniini**	*Arthropterus*	*A.* sp.	Q6, Q11	[34]
		*Mystropomus*	*M. regularis*	Q2, Q6, Q11	[34]
		*Pachyteles*	*P. longicornis*	H52, Q2	[59]
			*P. striola*	H52, Q2	[59]
		*Physea*	*P. hirta*	H52, Q2	[59]
		*Platycerozaena*	*P. panamensis*	H52, Q2, Q8, Q11	[59]
	**Paussini**	*Homopterus*	*H. arrawi*	H52, Q2	[59]
		*Paussus*	*P. favieri*	Q2, Q11	[92]
**Cicindelinae**	**Cicindelini**	*Cicindela*	*C. flexuosa*	E9, E19	[93]
			*C. haemorrhagica*	A1, B1, E19, H52, H62	[79]
			*C. marutha*	E9	[79]
			*C. nigrocoerulea*	E19	[79]
			*C. punctulata chihuahuae*	A1, E19	[79]
			*C. sedecimpunctata*	A1, T3	[79]
			*C. sexguttata*	T3	[79]
			*C. abdominalis, C. andrewesi,* *C. angulata, C. assamensis, C. aurofasciata, C. belfragei, C. bicolor, C. bigemina, C. calligramma, C. cancellata, C. cardoni, C. catena, C. celeripes, C. chloris, C. circumpicta, C. cuprascens, C. depressula, C. duodecimguttata,* *C. duponti, C. erudita, C. f. generosa, C. f. manitoba, C. fabriciana, C. fastidiosa, C. fowleri, C. fulgida, C. grammophora, C. hamata, C. hamiltoniana, C. hirticollis, C. horni, C. intermedia, C. lemniscata, C. limbata, C. macra,* *C. melancholica, C. minuta, C. motschulskyana, C. multiguttata, C. nevadica, C. o. rectilatera, C. obsoleta, C. ocellata ocellata, C. oregona,* *C. pamphila, C. pimeriana,* *C. pulchra, C. punctulata punctulata, C. purpurea, C. repanda, C. rufiventris, C. rugosiceps, C. s. lecontei,* *C. s. rugifrons, C. schauppi, C. severa, C. severini, C. striatifrons, C. striolata, C. sumatrensis, C. togata globicollis, C. tranquebarica, C. venosa, C. virgula, C. westermanni, C. willistoni, C. lengi*	A1	[79]
		*Odontocheila*	*O. annulicornis, O. cayennensis, O. confuse, O. luridipes*	A1, H52	[79]
		*Pentacomia*	*P. egregia*	A1, H52	[79]
**Cicindelinae**	**Collyridini**	*Neocollyris*	*N. variitarsus*	A1	[79]
	**Megacephalini**	*Megacephala*	*M. carolina*	A1, B8, N1	[79]
		*Omus*	*O. audouini*	B15	[79]
**Carabinae**	**Carabini**	*Calosoma*	*C. (Campalita) chinense*	C4, F27, A2	[33]
			*C. externuum*	C4	[28]
			*C. marginalis*	C4	[28]
			*C. oceanicum*	A2, C4, F5	[34]
			*C. prominens*	A2	[30,64]
			*C. schayeri*	A2, C4, F5	[34]
			*C. sycophanta*	A2, C4, C5, B1, F2, F6, F11, F17, F25, F27	[30,48]
		*Carabus*	*C. auratus*	C4, F27	[28,30,78,94]
			*C. auronitens*	C4, F27	[30,78,94,95]
			*C. (Damastes) blaptoides*	C2, C4, F27	[33]
			*C. (Megodontus) caelatus*	B1, C1, C4, F1, F2, F8, F11, F17, F25, F27	[43]
			*C. (Tachypus) cancellatus*	C4, F27	[30]
			*C. cansellatus*	C4, F27	[78]
			*C. cyaneus*	C4, F27	[30]
			*C. (Tomogarabus) convexus*	B1, C4, F27	[30,42,78,94]
			*C. (Procustes) coriaceus*	B1, C4, F27	[30,42,49,94]
			*C. (Apotomopterus) dehaanii*	C2, C4, F27	[33]
			*C. granulatus*	C4, F27	[30,78,94]
			*C. intricatus*	C4, F27	[94]
			*C. (Platycarabus) irregularis*	C4, F27	[30,94]
			*C. (Apotomopterus) japonicus*	C2, C4, F27	[33]
			*C. (Archicarabus) montivagus*	C4, F27	[43]
			*C. porrecticollis*	C2, C4, F27	[33]
			*C. problematicus*	C4, F27	[28,78]
			*C. procelus*	C2, C4, F27	[30,33]
			*C. taedutus*	C2, C4	[67]
			*C. ullrichii*	B1, C4, F1, F2, F11, F17, F27	[30,49,94]
			*C. (M.) violaceus*	B1, C4, F1, F11, F17, F25, F27	[30,43,94]
			*C. (Apotomopterus) yaconinus*	C2, C4, F27	[33]
		*Hemicarabus*	*H. tuberculosus*	C2, C4	[33]
**Carabinae**	**Ceroglossini**	*Ceroglossus*	*C. buqueti*	B1, C1, C2, C4, C5, F3, F11, F14, F27, H61, S1	[96]
			*C. chilensis*	B1, C2, C4, F3, F11, F14, F27, H61	[96]
			*C. magellanicus*	B1, C2, C4, F3, F11, S1	[96]
	**Cychrini**	*Cychrus*	*C. caraboides rostratus*	C4, F27	[28,30,94]
		*Scaphinotus*	*S. andrewsi germari, S. andrewsi montana, S. virdus, S. webbi*	C4, F27	[77]
	**Pamborini**	*Pamborus*	*P. alternans, P. guerini, P. pradieri, P. viridis*	C2, C4	[34]
**Elaphrinae**	**Elaphrini**	*Elaphrus*	*E. riparius*	F11, F14	[28]
**Loricerinae**	**Loricerini**	*Loricera*	*L. pilicornis*	F11, F14	[28]
**Omophroninae**	**Omophronini**	*Omophron*	*O. limbatum*	F11, F14	[28]
**Nebriinae**	**Nebriini**	*Leistus*	*L. ferrugineus*	C4, F27	[28,78]
		*Nebria*	*N. chinensis*	C2, C4, F27	[33]
			*N. lewisi*	C2, C4, F27	[33]
			*N. livida*	C4, F27	[28,78]
			*N. macrogona*	C2, C4, F27	[33]
			*N. psammodes*	C4, F27	[50]
**Nebriinae**	**Notiophilini**	*Notiophilus*	*N. biguttatus*	F11, F14	[28]
			*N. impressifrons*	C4, F27	[33]
**Scaritinae**	**Scaritini**	*Scarites*	*S. aterrimus*	C4, F1, F6, F13, F27	[33]
			*S. subterraneus*	C4, F1, F6, F11, F13, F27	[68]
			*S. cutidens*	C4, F1, F13, F27	[33]
			*S. sulcatus*	C4, F1, F13, F27	[33]
			*S. terricola*	C4, F1, F13, F27	[33]
	**Clivinini**	*Ardistomis*	*A. schaumii*	Q11, T1, T4	[75]
		*Clivina*	*C. basalis*	Q1, Q11	[34]
			*C. fossor*	Q1, Q6, Q2, Q11, Q12, Q13	[78]
		*Semiardistomis*	*S. puncticollis*	Q2, Q11, T2, T4, T5, T6, T7	[75]
		*Schizogenius*	*S. lineolatus*	F8, F9, F10	[31]
	**Dyschiriini**	*Dyschirius*	*D. wilsoni*	B9, K2, K7, T3	[97]
	**Pasimachini**	*Pasimachus*	*P. subsulcatus*	C4, F1, F11, F14, F17, F25, F27	[51,52]
	**Carenini**	*Carenum*	*C. bonelli*	C4, F1, F7, F13	[34]
			*C. interruptum*	C4, F1, F13	[34]
			*C. tinctillatum*	C4, F1, F13, F27	[34]
		*Laccopterum*	*L. foveigerum*	C2, C4, F5, F6, F7, F13, F27	[34]
		*Philoscaphus*	*P. tuberculatus*	C4, F6, F12, F13, F27	[34]
**Broscinae**	**Broscini**	*Broscosoma*	*B. doenitzi*	F2, F14	[33]
		*Broscus*	*B. cephalotes*	F11, F14	[28,98,99]
		*Craspedonotus*	*C. tibialis*	F1, F11, F14	[33]
		*Eurylychnus*	*E. blagravei*	C4, F27	[34]
			*E. ollifi*	C4, F17, F27	[34]
**Trechinae**	**Trechini**	*Duvalius*	*D. (Paraduvalius) milutini*	B1, F4, F5, F15, F18, F22, F23, F24, F26	[100]
		*Pheggomisetes*	*P. ninae*	A1, C1, C5, F2, F11, F12, F14, F16, F18, F22, F23, F26, H12, H16, H17, H24, H34, H38, H39, H40, H45, H48, H49, H62, H65	[100]
		*Trechoblemus*	*T. postilenatus*	F11, F14	[33]
	**Bembidiini**	*Bembidion*	*B. lampros*	F11, F14	[28]
			*B. lissonotum*	C4, F27	[33]
			*B. morawitzi*	C4, F27	[33]
			*B. quadriguttatum*	A2, F28	[78]
			*B. semilunium*	C4, F27	[33]
			*B. stenoderum*	C4, F27	[33]
		*Tachys*	*T. sericans*	F11, F14	[33]
**Patrobinae**	**Patrobini**	*Amblytelus*	*A. curtus*	C3	[34]
		*Asaphidion*	*A. semilucidum*	F2, F14	[33]
		*Diplous*	*D. caligatus*	C4, F27	[33]
			*D. depressus*	C4, F27	[33]
		*Patrobus*	*P. flavipes*	C4, F1, F13, F27	[33]
			*P. longicornis*	C1, C4, F27, K6	[31]
**Harpalinae**	**Morionini**	*Morion*	*M. simplex*	C1, C3, C4, F25, F27, H3, H4, H5, H6	[31]
		*Moriosomus*	*M. seticollis*	B1, C1, C3, E6, E19, E20, E21, H3, H5, H6, H7	[31]
	**Perigonini**	*Diploharpus*	*D. laevissimo*	C1, C3, E1, E3, E4, E12, H1, H3	[31]
	**Loxandrini**	*Loxandrus*	*L. icarus*	C1, C3, F7, H2, H3	[31]
			*L. longiformis*	A2	[34]
			*L. velocipes*	C1, C3, F7, H3, H7	[31]
	**Sphodrini**	*Calathus*	*C. fuscipes*	C3	[98,99]
		*Dolichus*	*D. halensis*	K8	[101]
		*Laemostenus*	*L. punctatus*	C1, C3, E6, E20, F22, F23, F26, G2, H30, H32, H65	[87,100]
		*Synuchus*	*S. callitheres*	C3	[33]
			*S. cycloderus*	C3, K8	[33]
			*S. dulcigradus*	C3, K8	[33,101]
	**Pterostichini**	*Abacomorphus*	*A. asperulus*	C3, C4, F1, F27	[34]
		*Abaris*	*A. anaea*	C4, F27, H3, H4, H5	[31]
		*Blennidus*	*B. liodes*	C4, F14, F27, H4	[31]
		*Castelnaudia*	*C. superba*	C1, C4, F27	[34]
		*Cratoferonia*	*C. phylarchus*	C4, F27	[34]
		*Cratogaster*	*C. melas*	C4, F27	[34]
		*Cyclotrachelus*	*C. sigillatus*	C1, C3	[31]
		*Gasterllarius*	*G. honestus*	C4, F25	[31]
		*Incastichus*	*I. aequidianus*	C1, C3	[31]
		*Lesticus*	*L. magnus*	C4, F27	[33]
		*Loxodactylus*	*L. carinulatus*	C3	[34]
		*Myas*	*M. coracinus*	C4, F25, F27	[31]
		*Notonomus*	*N. angustibasis, N. crenulatus, N. miles, N. muelleri, N. opulentus, N. rainbowi, N. scotti, N. triplogenioides, N. variicollis*	C3	[34]
		*Poecilus*	*P. coerulescens*	C4, F27	[33]
			*P. cupreus*	C4, F27, H8, H62, H65	[78]
			*P. fortipes*	C4, F27	[33]
		*Prosopogmus*	*P. harpaloides*	C4	[34]
		*Pseudoceneus*	*P. iridescens*	C4, F27	[34]
		*Pterostichus*	*P. (Hypherpes) californicus*	C2, F17, F27	[69]
			*P. (Cophosus) cylindricus*	C4, F27	[44]
			*P. daisenicus*	C2, C4, F27	[33]
			*P. (Monoferonia) diligendus*	C4, F27	[31]
			*P. externepunctatus* *roccai*	C4, F11, F27, H62, H65	[50]
			*P. fortis*	C2, C4, F27	[33]
			*P. fujimurai*	C2, C4, F27	[33]
			*P. longinquus*	C2, C4, F27	[33]
			*P. luctuosus*	C4, F27	[31]
			*P. macer*	C4, F27, H8, H62, H65	[28,78]
			*P. masidai*	C2, C4, F27	[33]
			*P. (Ferodinius) melas*	C4, F11, F17, F25, F27, H8, H62, H65	[28,44,78]
			*P. metallicus*	C4, F27, H8, H62, H65	[28,30,78,94]
			*P. microcephalus*	C2, C4, F27	[33]
			*P. niger*	C4, F27, H8, H62, H65	[28,30,78,94]
			*P. (Pseudomaseus) nigrita*	C1, C4, C5, F9, F11, F17, F27, H13, H14, H15, H57, H60, H62, H64, H65	[44]
			*P. prolongatus*	C2, C4, F27	[33]
			*P. rotundangulus*	C2, C4, F27	[33]
			*P. vulgaris*	C4, F27, H8, H62, H65	[30,78,94]
		*Rhytisternus*	*R. laevilaterus*	C4, F27	[34]
		*Sarticus*	*S. cyaneocinctus*	C3	[34]
		*Sphodrosomus*	*S. saisseri*	C3	[34]
		*Trichosternus*	*T. nudipes*	C4, F27	[34]
		*Trigonotoma*	*T. lewisii*	C4, F27	[33]
		*Trigonognatha*	*T. cuprescens*	C4, F27	[33]
	**Platynini**	*Agonum*	*A. chalcomum*	C3, K8	[33,101]
			*A. daimio*	C3	[33]
		*Anchomenus*	*A. (Idiochroma) dorsalis*	H14, H58, H60, H65	[28]
			*A. leucopus*	C3	[33]
		*Colpodes*	*C. atricomes*	C3	[33]
			*C. japonicus*	C3	[33]
		*Loxocrepis*	*L. rubriola*	C3	[33]
		*Lorostemma*	*L. ogurae*	C3	[33]
		*Platynus*	*P. brunneomarginatus*	C3, F18, F23, H52, H62, H65, K1, K3, K6, K8	[31]
			*P. magnus*	C3	[33]
			*P. ovipennis*	C3, F19, H52, H62, H65, K1, K3, K6, K8	[102]
			*P. protensus*	C3, K8	[33,101]
	**Zabrini**	*Amara*	*A. chalcites*	C2, C4, F1, F27	[33]
			*A. chalcophaea*	C2, C4, F1, F27	[33]
			*A. familiaris*	C4, F27, H8, H62, H65	[28,78]
			*A. similata*	C4, F27, H8, H62, H65	[28,78]
		*Bradytus*	*B. ampliatus, B. simplicidens*	C2, C4, F1, F27	[33]
		*Curtonotus*	*C. giganteus*	C2, C4, F1, F27	[33]
	**Molopini**	*Abax*	*A. ovalis*	C4, F27	[28,30,78]
			*A. parallelepipedus* *(*sub*:A. ater)*	C4, C5, F6, F11, F25, F27	[28,30,49,78]
			*A. parallelus*	C4, F27	[30,78,95]
		*Molops*	*M. elatus*	C4, F27	[28,78]
			*M. (Stenochoromus) montenegrinus*	C1, C4, C5, F1, F2, F5, F8, F9, F11, F17, F28	[44]
	**Harpalini**	*Anisodactylus*	*A. signatus*	C3, K8	[33,101]
			*A. tricuspidatus*	C3	[33]
		*Anoplogenius*	*A. cyanescens*	C3	[33]
		*Bradycellus*	*B. inornatus*	C3	[33]
		*Diaphoromerus*	*D. edwardsi*	C3	[34]
		*Harpalus*	*H. atratus*	C3	[98,99]
			*H. capito*	C3, K8	[33,101]
			*H. dimidiatus*	C3	[30]
			*H. distinguendus*	C3	[98,99]
			*H. luteicornis*	C3	[98,99]
			*H. platynotus*	C3	[33]
			*H. sinicus*	C3	[33]
			*H. tardus*	C3	[98,99]
		*Platymetopus*	*P. flavibarbis*	C3	[33]
		*Pseudophonus*	*P. griseus*	C3	[30,33,94]
			*P. rufipes (sub:pubescens)*	C3	[30,94]
		*Stenolophus*	*S. agonoides*	C3, H63	[33,101]
			*S. difficilis*	C3	[33]
			*S. iridicolor*	C3	[33]
		*Trichocellus*	*T. tenuimanus*	C3	[33]
		*Trichotichnus*	*T. longitarsis*	C3, H63	[33,101]
	**Licinini**	*Dicrochile*	*D. brevicollis*	C3	[34]
			*D. goryi*	C3	[34]
		*Diplocheila*	*D. elongata*	C3	[33]
			*D. zeelandica*	C3, H63	[33,101]
		*Syagonix*	*S. blackburni*	C3	[34]
	**Chlaeniini**	*Callistoides*	*C. delciolus*	B2	[33]
		*Callistus*	*C. lunatus*	Q2, Q11	[28]
			*C. basalis*	Q2	[28]
		*Chlaenius*	*C. australis*	B2	[34]
			*C. circumdatus*	B2	[33]
			*C. cordicollis*	B2, B4, B3, B5, B7, B11, B12	[30,91]
			*C. (Chlaeniellus) inops*	Q1, Q6, Q10	[33]
			*C. noguchii*	B2	[33]
			*C. pallipes*	B2	[33]
			*C. (Chlaeniellus) postemus*	Q1, Q6, Q10	[33]
			*C. spoliatus*	B2	[33]
			*C. velutinus*	B2, B4, B6, H22, H52, Q1, Q7, Q11	[50]
			*C. vestitus*	B2, Q2	[28,50]
			*C. virgulifer*	B2	[33]
		*Epomis*	*E. nigricans*	B2	[33]
		*Macrochlaenites*	*M. costiger*	B2	[33]
		*Oodes*	*O. amaroides*	A2, B1, C1, F11, F14	[31]
			*O. americanus*	B1, C1, C4, C5, F11, F2, F5, F6, F8, F14, F17, F21, F27	[89]
	**Panagaenini**	*Dischissus*	*D. mirandus*	B2	[33]
		*Panagaeus*	*P. bipustulatus*	B2	[98,99]
			*P. japonicus*	B2	[33]
		*Peronomerus*	*P. auripilis*	B2	[33]
			*P. nigrinus*	B2	[33]
	**Odacanthini**	*Archiocolliuris*	*A. bimaculata nipponica*	H63	[101]
		*Colliuris*	*C. pensylvanica*	C1, C3, H3, K6	[31]
		*Odacantha*	*O. melanura*	C3	[28]
	**Lebiini**	*Apristus*	*A. grandis*	C3, H63	[33,101]
		*Callida*	*C. lepida*	C3	[33]
		*Lebia*	*L. retrofasciata*	C3	[33]
		*Eudalia*	*E. macleayi*	C3	[34]
		*Coptodera*	*C. japonica*	C3	[33]
			*C. subapicalis*	C3	[33]
		*Cymindis*	*C. daimio*	H63	[101]
		*Dolichoctis*	*D. striatus*	C3, H63	[33,101]
		*Helluomorphoides*	*H. clairvillei*	H43, H9, H8, H65, E8, H11, E17, E14, E16, E12, E3, E15, E1, E21, E3, E22, E13, E5, E20, E7, E6, H14, H31, H58, H33	[85]
			*H. ferrugineus*	C3, E12	[84]
			*H. latitarsis*	C3, E12	[84]
		*Lebidia*	*L. octoguttata*	C3, H63	[33,101]
	**Galeritini**	*Galerita*	*G. lecontei*	C3, E1, E2, E3, E5, E6, E13, E12, E17, E20, G1, H8, H43, H44, H46, H62, H65, H66, H67	[45]
		*Galeritula*	*G. japonica*	C3, E1, E3, E12	[33,101]
		*Planetes*	*P. puncticeps*	C3, E1, E3, E12	[33,101]
	**Anthiini**	*Anthia*	*A. thoracica*	A3, C1, C3, F1, F27	[103]
		*Thermophilum*	*T. burchelli*	C1, C3, F1, F27	[103]
			*T. homoplatum*	A3, C1, C3, F1, F27	[103]
	**Helluonini**	*Helluo*	*H. costatus*	C3, E12, E13	[34]
	**Catapieseini**	*Catapiesis*	*C. attenuata*	C3, E1, H1, H3	[31]
			*C. sulcipennis*	E1	[31]
	**Dryptini**	*Drypta*	*D. japonica*	C3, E1, E3, E12	[33,101]
	**Pseudomorphini**	*Sphallomorpha*	*S. colymbeioides*	C3	[34]
**Brachininae**	**Brachinini**	*Brachinus*	*B. chuji*	Q1, Q9	[33]
			*B. crepitans*	Q2, Q11	[28,30]
			*B. elongatus*	H10, H14, H60, Q2, Q4, Q5, Q8, Q11, Q12	[41,55,66]
			*B. explodens*	Q2, Q11	[28,30]
			*B. sclopeta*	Q2, Q11	[30,41]
			*B. scotomedes*	Q1, Q9	[33]
			*B. stenoderus*	Q1, Q9	[33]
		*Pheropsophus*	*P. africanus*	N2, N3	[30,41]
			*P. agnatus*	C3	[30]
			*P. catoirei*	Q2, Q11	[104]
			*P. verticalis*	Q1, Q11	[34]
			*P. jessoensis*	Q1, Q9	[33]

* Abbreviations [**Aldehydes (A)**—**A1**: benzaldehyde; **A2**: salicylaldehyde; **A3**: iso-valeraldehyde. **Benzene, substituted derivatives and phenols (B)**—**B1**: benzoic acid; **B2**: cresols, (m-cresol), (3-methylphenol); **B3:** 2,3-dimethylphenol; **B4**: 2,5-dimethylphenol; **B5**: 3,4-dimethylphenol; **B6**: 3,5-dimethylphenol; **B7**: 3-ethylphenol; **B8**: mandelonitril; **B9**: methyl 2-hydroxy-6-methylbenzoate; **B10**: methyl salicylate; **B11**: 2-methoxy-5-methylphenol; **B12**: 2-methoxy-4-m-cresol; **B13**: 2-phenylethanol; **B14**: 2-phenylethyl; **B15**: phenylacetic acid; **B16**: xylenol isomer. **Carboxylic acids and derivatives (C)**—**C1**: acetic acid; **C2**: ethacrylic acid; **C3**: formic acid; **C4**: methacrylic acid; **C5**: propanoic acid (propionic acid). **Fatty alcohol esters (E)**—**E1**: decyl acetate; **E2**: decyl butyrate; **E3**: decyl formate; **E4**: decyl hexanoate; **E5**: decyl propionate; **E6**: dodecyl acetate; **E7**: dodecyl formate; **E8**: heptyl acetate; **E9**: hexadecyl acetate; **E10**: isopropyl ethacrylate; **E11**: isopropyl methacrylate; **E12**: nonyl acetate; **E13**: nonyl butyrate; **E14**: nonyl formate; **E15**: nonyl propionate; **E16**: 3-nonen-l-yl acetate; **E17**: octyl acetate; **E18**: 2-phenylethyl ethacrylate; **E19**: tetradecyl acetate; **E20**: undecyl acetate; **E21**: undecyl formate; **E22**: 4-undecen-l-yl acetate. **Fatty acids and conjugates (F)**—**F1**: angelic acid; **F2**: butyric acid; **F3**: n-butanoic acid; **F4**: capric acid; **F5**: caproic acid (hexanoic acid); **F6**: crotonic acid; **F7**: hexenoic acid; **F8**: 2-hexenoic acid; **F9**: 3-hexenoic acid; **F10**: 3,5-hexadienoic; **F11**: isobutyric acid; **F12**: isocaproic acid; **F13**: isocrotonic acid; **F14**: isovaleric acid (3-methylbutyric acid); **F15**: lauric acid; **F16**: linoleic acid; **F17**: 2-methylbutyric acid; **F18**: myristic acid; **F19**: nonanoic acid; **F20**: octanoic acid (caprylic acid); **F21**: 2-octenoic acid; **F22**: oleic acid; **F23**: palmitic acid; **F24**: pelargonic acid; **F25**: senecioic acid; **F26**: stearic acid; **F27**: tiglic acid; **F28**: valeric acid. **Fatty alcohol (G)**—**G1**: 1-decanol; **G2**: dodecan-1-ol. **Hydrocarbons (H)**—**H1**: C9:0; **H2**: C10:0; **H3**: C11:0; **H4**: C12:0; **H5**: C13:0; **H6**: C15:0; **H7**: C17:0; **H8**: decane; **H9**: 1-decene + 3-decene; **H10**: 9-docosene; **H11**: dodecane; **H12**: 3-ethyltetracosane; **H13**: heneicosadiene; **H14**: heneicosane; **H15**: heneicosene; **H16**: heptacosadiene; **H17**: heptacosene; **H18**: 5,7-heptadecadiene; **H19**: 7,9-heptadecadiene; **H20**: (6Z,9Z)-6,9-heptadecadiene; **H21**: (7Z,9Z)-7,9-heptadecadiene; **H22**: heptadecane; **H23**: (Z)-8-heptadecene; **H24**: hexacosane; **H25**: hexadecadiene; **H26**: 6,8-hexadecadiene; **H27**: 7,9-hexadecadiene; **H28**: hexadecane; **H29**: hexadecene; **H30**: 7-hexyldocosane; **H31**: 9-methylheneicosane; **H32**: 9-methyltetracosane; **H33**: 9-methyltricosane; **H34**: 11-methylheptacosane; **H35**: 3-methylpentadecane; **H36**: 4-methylpentadecane; **H37**: 5-methylpentadecane; **H38**: nonacosapentaene; **H39**: nonacosatetraene; **H40**: nonacosene; **H41**: nonadecane; **H42**: 7,9-nonadecadiene; **H43**: nonane; **H44**: 1-nonene; **H45**: octacosane; **H46**: octane; **H47**: pentacosadiene; **H48**: pentacosane; **H49**: pentacosene; **H50**: (Z)-7-pentacosene; **H51**: (Z)-9-pentacosene; **H52**: pentadecane; **H53**: 5,7-pentadecadiene; **H54**: 6,8-pentadecadiene; **H55**: 7-pentadecene; **H56**: tetradecane; **H57**: tricosadiene; **H58**: tricosane; **H59**: (Z)-7-tricosene; **H60**: (Z)-9-tricosene; **H61**: 11-tricosene; **H62**: tridecane; **H63**: 2-tridecane; **H64**: tricosatriene; **H65**: undecane; **H66**: 4-undecene; **H67**: 5-undecene. **Ketone (K)**—**K1**: 2-dodecanone; **K2**: 2-heptanone; **K3**: 2-heptadecanone; **K4**: 2-hexanone; **K5**: 3-hexanone; **K6**: 2-pentadecanone; **K7**: 2-pentanone; **K8**: 2-tridecanone. **Non-metal oxoanionic compounds and organonitrogen compounds (N)**—**N1**: hydrogen cyanide; **N2**: nitrites; **N3**: nitrous acid. **Quinone (Q)**—**Q1**: benzoquinone; **Q2**: 1,4-benzoquinone; **Q3**: 2-chloro-1,4-benzoquinone; **Q4**: 2,5-dimethyl-1,4-benzoquinone; **Q5**: 2,3-dimethyl-1,4-benzoquinone; **Q6**: 2-ethylquinone; **Q7**: ethylbenzoquinone; **Q8**: 2-ethyl-1,4-benzoquinone; **Q9**: 2-methylbenzoquinone; **Q10**: 2-methylquinone; **Q11**: 2-methyl-1,4-benzoquinone (toluquinone); **Q12**: methoxy-1,4-benzoquinone; **Q13**: 2-methoxy-3-methyl-1,4-benzoquinone. **Terpenes (T)**—**T1**: 1,8-cineole; **T2**: p-cymene; **T3**: iridodial; **T4**: (R)-(+)-limonene, (S)-(-)-limonene; **T5**: sabinene; **T6**: β-phellandrene; **T7**: β-pinene. **Thioethers (S)**—**S1**: 3-methyl-1-(methylthio)-2-butene.].

## Data Availability

No new data were created or analyzed in this study. Data sharing is not applicable to this article.
